# Lessons from Genome-Wide Search for Disease-Related Genes with Special Reference to HLA-Disease Associations

**DOI:** 10.3390/genes5010084

**Published:** 2014-02-26

**Authors:** Katsushi Tokunaga

**Affiliations:** Department of Human Genetics, Graduate School of Medicine, University of Tokyo, Tokyo 113-0013, Japan; E-Mail: tokunaga@m.u-tokyo.ac.jp

**Keywords:** genome-wide association study (GWAS), multifactorial disease, disease susceptibility gene, drug response gene, HLA genes

## Abstract

The relationships between diseases and genetic factors are by no means uniform. Single-gene diseases are caused primarily by rare mutations of specific genes. Although each single-gene disease has a low prevalence, there are an estimated 5000 or more such diseases in the world. In contrast, multifactorial diseases are diseases in which both genetic and environmental factors are involved in onset. These include a variety of diseases, such as diabetes and autoimmune diseases, and onset is caused by a range of various environmental factors together with a number of genetic factors. With the astonishing advances in genome analysis technology in recent years and the accumulation of data on human genome variation, there has been a rapid progress in research involving genome-wide searches for genes related to diseases. Many of these studies have led to the recognition of the importance of the human leucocyte antigen (HLA) gene complex. Here, the current state and future challenges of genome-wide exploratory research into variations that are associated with disease susceptibilities and drug/therapy responses are described, mainly with reference to our own experience in this field.

## 1. Development of Genome-Wide Searches

The greatest attraction of the strategy of genome-wide searches for genes related to diseases is the potential for the discovery of the involvement of completely new genes that could not have been predicted using existing knowledge or data. The previous method for genome-wide search of multifactorial disease-susceptibility genes was non-parametric linkage analysis, which does not presuppose any specific inheritance mode. One such method is the affected sib-pair method. However, it is not easy to collect a large number of samples with affected sib-pairs, so the detection power of this method is inevitably low [1]. Consequently, only limited results have been obtained so far.

The genome-wide association study (GWAS), however, makes use of the high statistical power of association analysis traditionally used for investigating the possible involvement of specific candidate genes, and applies it genome-wide [1]. Two pioneering GWAS studies were carried out in Japan. One was the first single nucleotide polymorphism (SNP)-based GWAS for myocardial infarction, which utilized an approximately 90,000 SNPs [2]. The other was the first microsatellite-based GWAS for rheumatoid arthritis, which used approximately 30,000 microsatellite polymorphisms [3]. However, only a few research groups adopted either of these platforms, due to the labor and cost they involved.

GWAS advanced to a new stage from 2006 onward, mainly as a result of two developments in infrastructure. The first was information infrastructure, typified by the Database of Single Nucleotide Polymorphisms (dbSNP) [4], the International HapMap Project [5] and the 1000 Genomes Project [6], which gathered together a vast range of information of genome variation that spanned the entire human genome. The other development was in technology infrastructure; this was the commercial release of platforms that allowed the analysis of several thousands of samples performed on several hundreds of thousands of SNPs and could be carried out relatively easily. The application of these developments meant that SNP-based GWAS became a broad-based, practical strategy, and in 2007, several studies were published from large-scale collaborations between multiple institutions. The subsequent rush to discover gene polymorphisms associated with different diseases or traits using GWAS was dramatic, and over 1600 types of significant associations with 250 diseases or traits have been reported [7]. Nevertheless, attention should be paid for GWAS in ethnically diverse populations, since the genome-wide SNP typing chips have been designed based on mainly European data, these chips may have limited utility in certain populations.

## 2. Identified Susceptibility Genes to Multifactorial Diseases

### 2.1. Population Differences in Disease Susceptibility Genes

A disease for which GWAS have shown striking results is type II diabetes. In 2007, several groups from Europe and North America reported results from different GWAS on several thousand patients and controls [8,9,10,11]. Over 11 susceptibility loci were identified, and over half of these were newly discovered. The following year, two independent groups from Japan reported a new susceptibility gene, *KCNQ1* [12,13]. Table 1 shows a comparison between European and Japanese populations of the allele frequency, odds ratio and *p*-value of *TCF7L2*, the most important susceptibility gene found in European populations, and *KCNQ1*, which was discovered in Japanese.* TCF7L2* showed a *p*-value of 10^−48^ in European populations, indicating a definite association with type II diabetes [8]. Among Japanese, however, the *p*-value is at a level of no more than 10^−4^ [14]. The main reason for this is the difference in minor allele frequency, which is lower in Japanese by an order of magnitude. Consequently, although the odds ratio is similar to European populations, no clear association was observed in an analysis of 2000 patients and 2000 healthy controls. A contrasting relationship can be seen with *KCNQ1* [12]. The *p*-value for Japanese samples was 10^−29^, indicating a definite association with type II diabetes, and the same clear association was found for Korean and Chinese samples. However, although European samples showed the same tendency of the odds ratio, the *p*-value was at a level of no more than 10^−4^.

**Table 1:** Population differences of susceptibility genes to type II diabetes.

Gen (SNP)	Population	Odds Ratio	*p*	Minor Allele Frequency
*TCF7L2* (rs7903146)	European [8,9,10,11]	1.37	1.0 × 10^−48^	0.31/0.25
*TCF7L2* (rs7903146)	Japanese [14]	1.70	7.0 × 10^−4^	0.05/0.02
*KCNQ1* (rs2237892)	European [12]	1.29	7.8 × 10^−4^	0.03/0.05
*KCNQ1* (rs2237892)	Japanese [12]	1.43	3.0 × 10^−29^	0.31/0.40

In other words, the main type II diabetes-susceptibility genes for European and East Asian populations, respectively, are, in fact, shared susceptibility genes by both populations, but because they differ greatly in frequency, their contribution in each respective population is different.

Several genetic factors, in addition to environmental factors, such as stress, are involved in the onset of narcolepsy, one of the hypersomnia. In the past, the only gene well established as a genetic factor for narcolepsy was *HLA-DR/DQ* [15]; then, we carried out a GWAS to search for new genetic factors [16]. As a result, an SNP located between *CPT1B* and *CHKB* on Chromosome 22 was found to be associated with narcolepsy. Japanese and Koreans were found to have similar allele frequency and both showed a significant association. However, although the odds ratio showed similar trends in European Americans and African Americans, we could not find a significant difference association, because of the low frequency of the susceptibility allele. We have also experienced significant population differences in other diseases, including tuberculosis [17], rheumatoid arthritis [18], glaucoma [19] and primary biliary cirrhosis [20].

The above diseases serve as examples of different contributions of multiple genetic factors in each population. Consequently, the study of each individual population would be essential to build a complete picture of the important genetic factors to complex diseases in the various human populations.

### 2.2. Susceptibility Genes Common to Different Diseases

There has been an increase in the number of reports of genetic factors that are common to different diseases. *GPC5* (glypican-5) has been found to be a susceptibility gene common to nephrotic syndrome diseases, such as membranous nephropathy, immunoglobulin A nephropathy and diabetic nephropathy (Table 2) [21]. We further confirmed the expression of the GPC5 protein in the glomerular podocytes and showed that the risk allele is associated with a high level of GPC5 expression.

**Table 2:** Common susceptibility gene *GPC5* (glypican 5) for acquired nephrotic syndrome [21].

Panel	Case: Minor Allele Frequency	Control: Minor Allele Frequency	*p* *	Odds Ratio
1	0.237	0.167	5.8 × 10^−3^	2.33 (1.25–4.35)
2	0.195	0.159	2.0 × 10^−5^	3.44 (1.89–6.25)
3	0.224	0.174	8.7 × 10^−6^	2.39 (1.61–3.55)
Combined	0.219	0.168	6.0 × 10^−11^	2.54 (1.91–3.40)

* Based on the recessive model of the minor allele (GG + GA *vs*. AA).

Meta-analysis of the largest-scale GWAS in Japan on rheumatoid arthritis (RA) led to the discovery of susceptibility genes that are common to various different autoimmune disorders [18]. The GWAS was performed on approximately 4000 patients and 17,000 controls, and a replication study was carried out with 5000 patients and 22,000 controls. In addition to previously reported susceptibility genes, nine new susceptibility genes were discovered. Among these are several susceptibility genes that have been also reported for systemic lupus erythematosus (SLE) and Graves’ disease.

Another example in our recent experience was primary biliary cirrhosis [20]. We performed a GWAS by a nation-wide collaboration; as a result, we discovered two new susceptibility genes. Interestingly, one of these, *TNFSF15*, has also been reported as a susceptibility gene for inflammatory bowel disease, including Crohn’s disease and ulcerative colitis. There are numerous other reports of genetic factors that are found to be common to various autoimmune and inflammatory diseases [22,23].

The presence of common susceptibility genes for different diseases suggests that at least part of the pathogenic mechanism of these diseases is shared. These results may contribute to the elucidation of the pathogenic mechanism of these diseases and to the development of new therapies.

### 2.3. Towards the Understanding of Pathogenic Mechanisms

As mentioned earlier, the new narcolepsy-susceptibility region, *CPT1B/CHKB*, was discovered through a GWAS performed to search for genetic factors other than the established factor, *HLA* [16]. Subjects possessing the risk allele of the susceptibility SNP showed significantly lower levels of mRNA expression of both *CPT1B* and *CHKB*. We also observed that narcolepsy patients show abnormally low levels of carnitine [24], on which *CPT1B* (carnitine palmitoyltransferase 1B) is relevant, and that carnitine improves the sleep of the patients [25]. Carnitine is known as the transporter of long-chain fatty acids into mitochondria, thus playing a crucial role in energy production.

Moreover, the new susceptibility gene, *TRA* (T cell receptor α), was discovered through a GWAS performed by a joint international research group [26]. SNPs located in the J region of *TRA* showed significant associations with narcolepsy in European and Asian populations. *TRA* and *HLA* are key molecules in the regulation of immune response in the acquired immunity. The same joint international research group also found that a polymorphism of *P2RY11*, which is also involved in the regulation of the immune system, is associated with narcolepsy [27]. From these results, it may be assumed that narcolepsy onset has at least two mechanisms: both autoimmunity to orexin (hypocretin)-producing cells and a disorder of fatty acid β-oxidation.

If we appreciate that multiple susceptibility genes that have been discovered belong to specific pathways or networks, they will provide useful hints toward clarifying the mechanism of disease onset or disease formation and also developing new drugs.

## 3. Identified Response Genes to Drugs/Therapies

### 3.1. Development of New Gene Tests

**Figure 1:**
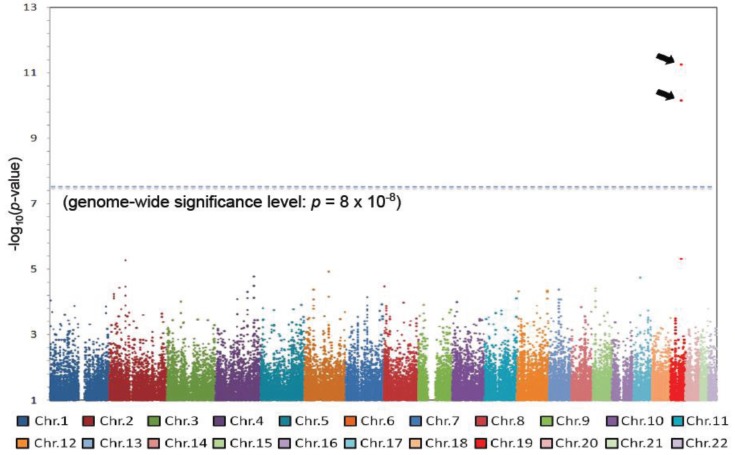
A genome-wide association study (GWAS) on the response to the combined therapy of PEGylated interferon-alpha and ribavirin for chronic hepatitis C identified two SNPs on Chromosome 19 [28].

**Figure 2:**
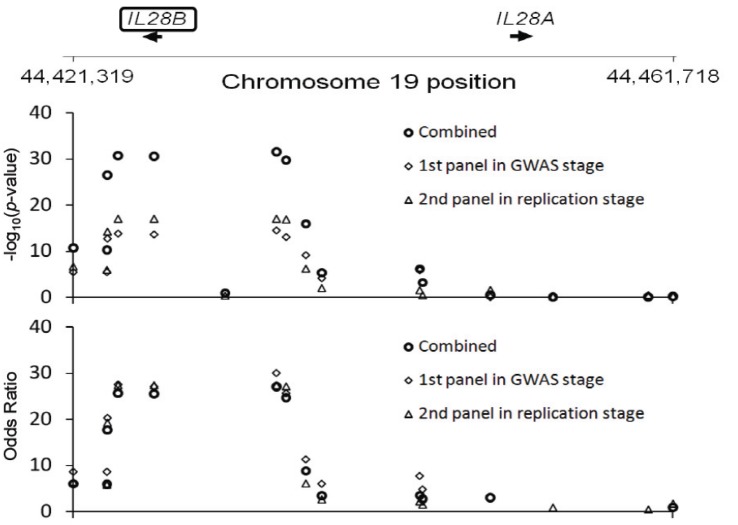
The strong association of *IL28B* with therapy response for chronic hepatitis C: 80% of non-responders possess the minor allele [28].

GWAS studies are extremely useful in the search for drug-response genes. We performed a GWAS as part of a multi-institutional joint research group investigating hepatitis C virus related diseases. As a result of this GWAS, we discovered that *IL28B* on Chromosome 19 was strongly associated with non-responder patients to the combined therapy of PEGylated interferon-alpha and ribavirin for chronic hepatitis C [28]. This was a completely unexpected result. The GWAS was performed on only 78 non-responders and 64 responders to this therapy; nevertheless a *p*-value at the level of 10^−12^ was obtained, reaching the genome-wide significance level (Figure 1). About 70%–80% of the non-responding patients possessed the minor alleles of several SNPs in the *IL28B* region, and combining the replication study data, the *p*-value was 10^−27^–10^−32^ and the odds ratio was 17−30 (Figure 2).

Response to the interferon-alpha therapy had been considered to be determined mainly by the virus genotype and concentration. However, the discovery that response is, in fact, mostly determined by a human genetic factor had a major impact. *IL28B* SNP typing has already been introduced into the routine clinical testing in Japan and is used as important reference data in the determination of therapeutic strategies.

### 3.2. Identification of New Therapeutic Targets

The discovery of *IL28B*, which is strongly associated with response to treatment for hepatitis C, indicated another highly interesting possibility. *IL28B* is a member of the interferon λ family and is assumed to exhibit its defensive activity against viral infection mediated by similar receptors and intracellular signal transduction pathway as interferon α, which was used in the treatment of hepatitis C. *IL28B* itself is therefore expected to be a powerful contender for the development of new hepatitis C drugs. In fact, *IL-29*, a member of the same family, has already been subjected to clinical trial for a new drug.

In addition to the above, genes involved in response to many drugs have been reported, and an increasing number of genetic factors are being identified for the first time as a result of GWAS. Drug-response genes generally tend to show greater odds ratios than disease-susceptibility genes, so that even with a relatively small sample size, there is a high likelihood of being able to identify the relevant gene. Ever greater results may therefore be expected in the future.

## 4. Particular Importance of HLA

### 4.1. Immune-Mediated Diseases and HLA

GWAS studies have been conducted for a number of diseases to date, and many of these have reported *HLA* as a susceptibility gene. In our own experience, narcolepsy [16], hepatitis B [29], rheumatoid arthritis [18], primary biliary cirrhosis [20], Stevens–Johnson syndrome, insulin autoimmune syndrome and type I diabetes have all shown strong association with certain *HLA* gene(s). Of these, narcolepsy, rheumatoid arthritis, primary biliary cirrhosis, type I diabetes and insulin autoimmune syndrome were associated most strongly with the *HLA-DR* and *HLA-DQ* regions, while hepatitis B and Stevens-Johnson syndrome were associated most strongly with the *HLA-DP* and *HLA-A* genes, respectively.

**Figure 3:**
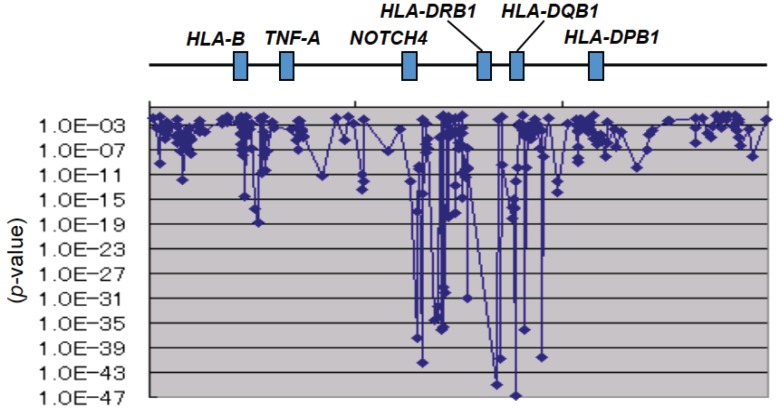
GWAS confirmed the most strong association of the *HLA-DR/DQ* region with narcolepsy [16].

With regard to narcolepsy, Juji *et al*. [30] first reported in 1984 an extremely strong association with *HLA-DR2* (HLA-DRB1*1501-DQB1*0602 haplotype according to the recent sequence-level nomenclature). We also found an extremely strong association between narcolepsy and the *HLA-DR/DQ* region with an SNP-based GWAS (Figure 3) [16]. If the results of HLA analysis in European and African populations are considered together, the primary susceptibility allele is assumed to be HLA-DQB1*0602.

Numerous GWAS have also been carried out for rheumatoid arthritis in Japan and elsewhere, and the *HLA-DR/DQ* region has been shown to have stronger association than any other region of the genome [18]. *HLA-DR4* has been known to be strongly associated with rheumatoid arthritis since the latter half of the 1970s; recent analysis at the sequence level has shown that DRB1*0401 is most strongly associated in European populations and DRB1*0405 among Japanese. However, there are several other *DRB1* alleles that also exhibit susceptibility or resistance, and a hierarchy may be seen in their odds ratios.

With primary biliary cirrhosis, also, the *HLA-DR/DQ* region showed the strongest association in the GWAS of European populations [31] and in the first GWAS of an Asian population [20]. From the analysis of *HLA* itself, HLA-DRB1*0803-DQB1*0602 and HLA-DRB1*0405-DQB1*0401 have been reported as susceptible haplotypes in the Japanese population [32], while HLA-DRB1*0801-DQB1*04 was reported in European descendants [33].

### 4.2. Drug Hypersensitivity and HLA

There has also been great interest in *HLA* in its association with drug hypersensitivity. In 2002, it was reported that nearly 80% of patients who showed a hypersensitivity against the HIV drug, abacavir, possessed HLA-B*5701, with an odds ratio of 117 [34]. In 2004, a group from Taiwan found that of 44 patients with Stevens–Johnson syndrome induced by carbamazepine used for epilepsy seizures or as a psychotropic drug, all had HLA-B*1502 [35]. However, less than 0.1% of Japanese possess HLA-B*5701, while HLA-B*1502 is extremely rare. Consequently, it was predicted that the associations observed in the previous reports are hardly seen at all among Japanese.

In fact, Ozeki *et al.* [36] reported that adverse reactions in the skin as a result of carbamazepine are associated with HLA-A*3101. We reported independently that Stevens–Johnson syndrome/toxic epidermal necrolysis accompanied by eye manifestations caused by certain types of cold remedies is associated with HLA-A*0206 [37]. Now, GWAS for this type of Stevens-Johnson syndrome has identified new susceptibility gene(s). Accordingly, GWAS can be powerful tool to investigate hypersensitivity to different kinds of drugs, and there is particular interest in associations with the *HLA* gene complex.

### 4.3. Characteristics of HLA and the Importance of HLA Typing

There are a number of unique characteristics of *HLA* genes and their polymorphisms, which indicates the limitation of SNP-based analysis and the importance of typing *HLA* genes themselves. First, the *HLA* genes are broadly classified into the Class I and Class II genes. Genes that exhibit high degrees of polymorphisms include *HLA-A*, *-B* and *-C* in Class I and *HLA-DRB1*, *-DQA1*, *-DQB1*, *-DPA1*, and *-DPB1* in Class II. Including *HLA* and non-HLA genes, a total of some 130 genes encoding proteins are densely located within a physical distance of about 4 Mbp on the short arm of Chromosome 6. They also show stronger linkage disequilibria than any other region of human genome. For these reasons, specifying a gene locus that is primarily associated with a disease is no easy task.

Second, commercially available genome-wide SNP typing arrays are unable to analyze the SNPs of the *HLA-DR* region. This is because there is copy number polymorphism of the *DRB* genes in the region: there are four functional *DRB* genes (*DRB1*, *B3*, *B4* and *B5*) and five pseudogenes*(DRB2*,* B6*,* B7*,* B8* and *B9*), and the gene composition differs depending upon the *DRB* haplotype. The SNPs of this region therefore do not conform to the Hardy–Weinberg equilibrium and, so, are not included on the arrays. Consequently, even though the *HLA-DQ* region may appear to show primary association from the results of an SNP-based GWAS, the adjacent *HLA-DR* region with extremely strong linkage disequilibrium must also be considered as a candidate region.

Third, genes in the Class II region are each adjacent on the genome as a pair, comprising an A gene and a B gene, and are linked to each other with a strong linkage disequilibrium. It is therefore very difficult to specify which gene of the pair is the primary one.

Fourth, as mentioned above, the *HLA* gene exhibits a high degree of polymorphism, and there are a huge number of alleles. There are almost no SNPs or SNP haplotypes that correspond one-on-one to individual *HLA* alleles. For example, more than 1300 alleles of *HLA-DRB1* have been admitted worldwide to date; for example, around 20 alleles with relatively high frequency and a great number of rare alleles have been found in the Japanese population; however, this sort of subclassification is not possible from SNP haplotypes.

Furthermore, a major feature is that a striking diversity between different populations can be observed. In other words, many *HLA* alleles are distributed only in certain regional populations.

Imputation of *HLA* alleles using *HLA* region SNP data is reported to have an accuracy of over 94% in European populations [38,39,40]. However, it is not perfect, especially for infrequent alleles, and the imputation is not yet fully available in Japanese or other Asian populations. The typing of the *HLA* genes is preferable for specifying *HLA* alleles directly involved in susceptibility, because there are multiple susceptibility alleles and resistance alleles, as well as ‘neutral’ alleles, and for many of these, the odds ratios are not consistent.

With regard to the *HLA*-associated diseases, therefore, detailed analysis, including the typing of the *HLA* genes themselves, are necessary to identify the primary *HLA* genes and alleles for each individual disease. These data will prove invaluable in clarifying the molecular mechanism through which HLA is associated with disease.

## 5. Conclusions and Issues for the Future

There are two hypotheses regarding the involvement of genome variation in common diseases: the common disease (common variants hypothesis and the common disease) and the rare variants hypothesis. In this regard, there is the argument that the common variants identified by GWAS as causing susceptibility to multifactorial diseases can only account for a small proportion of the genetic factors of disease, so that rare variants must also be important. This was symbolized by the term “missing heritability” [41], when only around 20 susceptibility loci for type II diabetes had been identified. Even in total, these could only explain about 5% of heritability. To date, over 60 common susceptibility loci have been identified, and this number is increasing all the time as a result of GWAS and meta-analyses carried out on greater scales. Further, it has been shown by the latest statistical analysis using all the GWAS data that around 40%−60% of all genetic factors can be explained. Therefore, it is assumed that there are still a great many relatively weak common susceptibility variants that have yet to be discovered.

To put it differently, we have not yet utilized the data obtained from GWAS to the fullest extent. For example, susceptibility genes that are not discovered by gathering samples from patients with the same disease name may be discovered by collecting detailed clinical data for each patient and then carrying out an analysis focused on clinical subsets. Considering a common disease from the viewpoint of its genetic architecture, the disease could be a collection of the many diseases that resemble each other, but also exhibit heterogeneity. Furthermore, it is likely that many susceptibility gene polymorphisms do not reach the so-called genome-wide significance level and, instead, exhibit moderate *p*-values. Establishing a method to identify the real susceptibility loci from this gray area is an issue that will need to be resolved in the future. It will be necessary to develop new methods that synthesize data from genetic ontology, pathway/network informatics and other fields and to establish statistical methods that can detect both intra-gene and inter-gene interactions. Our collaborators developed one such method that greatly improves the detection power of susceptibility loci [42].

Other than investigation by means of SNPs, there is also a need to clarify the degree to which variation, such as copy number variation (CNV) and short insertion/deletion variation, account for genetic factors in disease. Massive sequencing using next-generation sequencers is leading to astounding developments; to date, it has been very useful in identifying single genes responsible for hereditary diseases, and it has recently started to be applied to the search for susceptibility genes of multifactorial diseases. Until now, exome analysis has not turned up major results with respect to multifactorial disease. Considering that the majority of susceptibility SNPs identified by GWAS have been discovered in regions that regulate gene expression rather than in regions that code proteins, large-scale whole genome sequencing with a large number of patient and control samples may be needed. Then, the major challenge for the future is to establish a system to extract valuable data from the huge data produced by this new technology and to detect variants associated with certain multifactorial diseases.

*HLA* is already essential in clinical testing, such as organ and bone marrow transplantation and platelet transfusion. In addition, its association with over 100 types of diseases, including various autoimmune and inflammatory disorders, as well as infectious diseases, has been reported since the 1970s. Research aimed at understanding the mechanism of *HLA*-disease association commenced in the 1980s, but even now, the mechanism is not clearly known. In the 1990s, also, researchers carried out many analyses of antigenic peptides eluted from *HLA* molecules prepared from mass cultured cells and analyses of T-cell clones created from patient samples, but were unable to gain a complete understanding of pathogenic peptides or the mechanisms of disease onset. It is hoped that there will be breakthroughs in the search for solutions to the huge riddle of disease mechanisms through advances, such as the diversity analysis of each *HLA* haplotype using next-generation sequencers, expression analysis of each *HLA* molecule using the latest protein chemistry and high-order structure analysis of the *HLA*-antigenic peptide-T-cell receptor complex.

Finally, the sharing of a huge amount of data produced by genome-wide variation analyses on various diseases through public databases, such as the Database of Genotypes and Phenotypes (dbGaP) [43], European Genome-Phenome Archive (EGA) [44] and GWAS Central [45], is crucial for the promotion of the complete identification of disease susceptibility genes and the understanding of the molecular mechanism of disease onset. We have also developed a public database for studies on the Japanese population [46,47,48].
